# An unusual presentation of an atypical hangman’s fracture

**DOI:** 10.4103/0974-2700.66537

**Published:** 2010

**Authors:** Fevzi Yilmaz, Sami Akbulut, Ozkan Kose

**Affiliations:** 1 Department of Emergency Unit, Diyarbakir Education and Research Hospital, Diyarbakir - 214 00, Turkey; Department of Surgery, Diyarbakir Education and Research Hospital, Diyarbakir - 214 00, Turkey; 2 Department of Orthopaedics and Traumatology, Diyarbakir Education and Research Hospital, Diyarbakir - 214 00, Turkey

The term “hangman’s fracture” is used in most studies to designate injury to the laminae, articular facets, body, pedicles, or pars interarticularis of the axis (C2). hangman’s fracture of the axis was classified initially by Effendi *et al*. and modified by Levine and Edwards as type I, II, IIA, and III. The mechanism of a hangman’s fracture: type I injuries result from hyperextension-axial loading force; type II injuries from an initial hyperextension–axial loading force followed by severe flexion; type IIA injuries from flexion-distraction; and type III injuries from flexion–compression.[1–6] Type IIA hangman’s fracture, so-called atypical Hangman’s fracture, is very rare and there are a few cases presented in the literature.

The optimal therapy for hangman’s fractures is still controversial. Types I and II can be treated conservatively with either a cervical collar or a halo traction, whereas type IIA and III fractures are to be treated with rigid immobilization.[1–3] Neurologic deficits are present in up to 33% of rare atypical hangman’s fractures, vs 6.8% of other types. We present a case diagnosed as an atypical hangman’s fracture.

**Figure 1:**
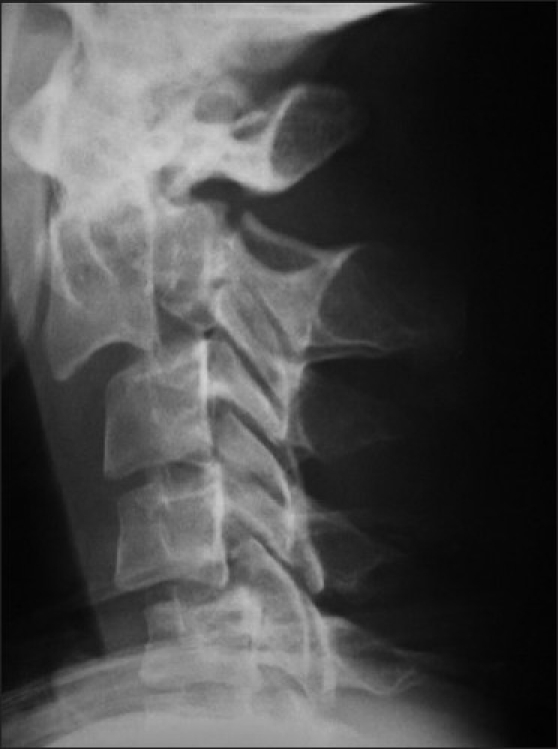
Radiography showed dens fracture on C2 (type III hangman’s fracture)

**Figure 2:**
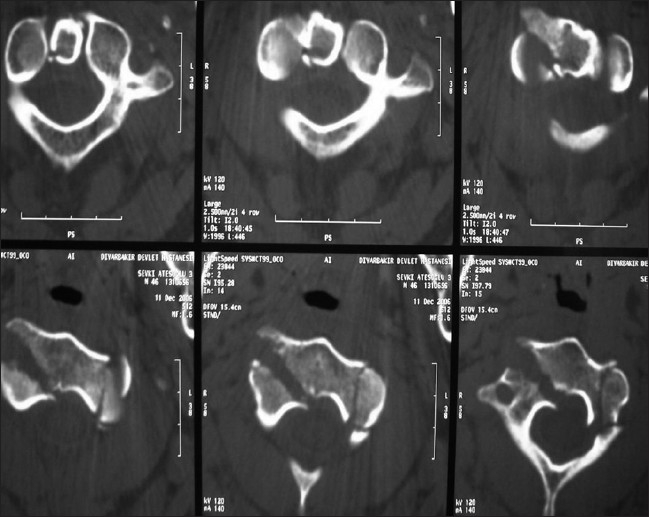
CT scan showed fracture of left pars interarticularis involving body of C2

A 46-year-old man was brought to the emergency room of a government hospital after a motor vehicle accident. He reported numbness in his right arm and upper cervical region. A neurologic examination revealed hypoesthesia and hemihypoalgesia between the C3 and C6 dermatomes, and the muscle strength in the upper right extremity was 4/5. Glasgow Coma Scale was 15. A lateral cervical radiograph showed C2 spondylolisthesis on C3 [Figure 1]. Cervical computed tomography (CT) revealed an axis fracture, including facet joint, lamina, and dens fractures, and was interpreted as an atypical hangman’s fracture (type IIA) [Figure 2]. After stabilization, the patient was transferred to the neurosurgery department of a university hospital, where he was treated surgically. The patient recovered fully by the end of the third postoperative week.

The most common cause of blunt trauma to the neck is motor vehicle accidents.[2] As almost half of spinal injuries result in neurologic deficits, we suggest stabilization of the cervical spine using a cervical collar for victims of motor vehicle accidents. A hangman’s fracture can be overlooked when other injuries are present, and hasty, unorganized efforts at resuscitation may worsen the fracture, possibly causing neurologic deficits. In addition, the traction applied when stabilizing the cervical spine may correct the malalignment, such that no signs of the fracture appear on radiographs. The high occurrence of neurologic deficits, such as paralysis, in atypical cases is explained by the continuity of the fracture in the posterior aspect of the vertebral body through the posterior cortex or pedicle, unilaterally or bilaterally. The fracture pattern and the degree of subluxation narrow the spinal canal, ultimately causing spinal cord injury.

Misdiagnosis of hangman’s fracture may lead to permanent damage to the spinal column. In patients with other injuries, such as tracheal rupture,[4] the chances of misdiagnosis are greater, because resuscitation attempts might have worsened the fracture, making traction and stabilization of the fracture impossible and leaving the patient exposed to additional cervical injury.[4]

The initial radiographs of the injured neck should include standard anteroposterior, lateral, and right, and left oblique projections.

A simple cervical spine lateral radiograph can diagnose the majority of the cases. However, using only radiography may lead to misdiagnosis. Certain types of injuries may remain occult on plain radiographs. Clinical suspicion and physical examination findings warrant further imaging investigations. In this respect, CT can be used to clarify questionable findings on plain radiographs, to reveal an otherwise occult injury, and to further evaluate a known fracture or fracture–dislocation. CT provides excellent bony detail of the fracture and degree of compromise of the spinal canal.[1–6]

Although routine radiography is vital for initial evaluation, CT is the gold standard for defining the fracture and guiding the treatment strategy. Unfortunately, CT may not be readily accessible in resource-limited settings. Therefore, mechanism of injury, clinical examination, and initial radiography findings are crucial to guide in ordering further imaging investigations. Similarly, in this presented case, especially the presence of neurologic deficit and lateral cervical radiography findings made us to order CT scan of the cervical spine. We could clearly indentify the injury pattern and classified the fracture as type IIA. Furthermore, in polytrauma patients, other injuries, such as possible head, thorax, and abdominal trauma, may mask the pain in the neck. Therefore, we suggest cervical CT along with cervical radiography in all motor vehicle accident victims with neurologic symptoms, polytrauma patients, and unconscious subjects.

In summary, atypical hangman’s fracture in motor vehicle accidents, suicide victims, and judicial cases commonly results from hyperextension and distraction of the upper cervical spine. Radiologic imaging techniques are very important for early diagnosis and management of hangman’s fracture.
